# Bounded rationality in healthcare: unraveling the psychological factors behind patient satisfaction in China

**DOI:** 10.3389/fpsyg.2024.1296032

**Published:** 2024-03-28

**Authors:** Yu Qian, Xiaohe Wang, Xianhong Huang, Jinwen Li, Chen Jin, Jie Chen, MengYi Sha

**Affiliations:** ^1^Department of Health Policy and Management, School of Public Health, Hangzhou Normal University, Hangzhou, China; ^2^Affiliated Hangzhou First People’s Hospital, School of Medicine, Westlake University, Hangzhou, China; ^3^The First Affiliated Hospital, Zhejiang University School of Medicine, Hangzhou, China

**Keywords:** patient satisfaction, bounded rationality theory, psychological factors, structural equation model, suppressive effect

## Abstract

**Introduction:**

Patient satisfaction is a crucial metric to gauge the quality of medical services, but the psychological factors influencing patient satisfaction remain insufficiently explored.

**Methods:**

This study examines these psychological factors by applying the theory of bounded rationality to 1,442 inpatients in Hangzhou, China, whose data were collected using a questionnaire. One-way ANOVA, correlation analysis, and hierarchical regression were used to analyze patient satisfaction and its associated factors. Additionally, the path analysis of the structural equation model revealed the mechanisms behind the key psychological factors that influenced patient satisfaction.

**Results:**

Medical risk perception, the social cognition of the medical environment, and social desirability bias had significant positive impacts on patient satisfaction. By contrast, negative emotions had a significant negative impact on patient satisfaction. Notably, patients’ negative emotions had both a suppressive effect and a positive moderating effect on the relationship between medical risk perception and patient satisfaction. Similarly, social desirability bias had a suppressive effect on the correlation between the social cognition of the medical environment and patient satisfaction, albeit with a negative moderating effect.

**Discussion:**

These results suggest that when evaluating and improving patient satisfaction, accounting only for the factors that directly influence medical service quality is insufficient, as the indirect and moderating effects of patients’ negative emotions and the social cognition of the medical environment must also be considered. Medical service providers should thus address patients’ negative emotions, establish good doctor–patient relationships, optimize service environments, provide managers with medical risk education and training on negative emotions, and prioritize patient-centered care. Additionally, the government and relevant health departments should optimize medical policies, enhance fairness and accessibility, and create a positive social cognitive environment through public education and awareness campaigns.

## Introduction

Patient satisfaction refers to the degree of consistency between the medical services patients expect and those they receive (Hooker et al., 2019; Ferreira et al., 2023). It serves as a vital indicator of a hospital’s fulfillment of its social duties, internal management, and service effectiveness (Asamrew et al., 2020; Versluijs et al., 2023). Owing to the increased attention paid to the quality of medical services, patient satisfaction has gained global recognition (Black et al., 2021; Grasso et al., 2021). It plays a pivotal role in promoting medical service improvements, enhancing the reputation of medical institutions, and formulating healthcare policy. Furthermore, patient satisfaction is deeply intertwined with the psychological and physical changes patients undergo due to illness, significantly altering their body image and self-perception (Sebri et al., 2022). This dynamic, along with the crucial role of social support in patient care (Durosini et al., 2021), emphasizes the need for a more holistic approach to understanding and measuring patient satisfaction.

Contemporary perspectives must be integrated into current theoretical frameworks, empirical analyses, and evaluation tools related to patient satisfaction (Hamasaki, 2022; Radwan et al., 2023). The consumer satisfaction theories (Afrashtehfar et al., 2020) that provided the initial framework for patient satisfaction have gradually incorporated a service quality model and expectancy confirmation theory to align themselves more closely with the characteristics of medical services (Oh et al., 2022; Ali et al., 2023). However, these theories often fall short in real-world applications, particularly by failing to capture the emotional and psychological complexities that patients experience during medical interactions (Bull et al., 2019; Banda et al., 2023). Numerous empirical studies have explored the factors influencing patient satisfaction, including medical service quality, doctor–patient relationships, and patient expectations (Asif et al., 2019; Abu-Rumman et al., 2022). While a consensus on these factors remains elusive, such research has significantly deepened our understanding of patient satisfaction, enriching the related theoretical frameworks and refining evaluation tools. For example, traditional questionnaires have been replaced with modern data science techniques that provide significantly more accurate and stable satisfaction assessments (Barsom et al., 2020; Hajesmaeel-Gohari et al., 2022). However, these tools can still fail to reflect patients’ true feelings and be inaccurate when used in certain survey environments or in tandem with some analysis methods (Dunsch et al., 2018). Thus, further research on these techniques and how to improve them is necessary.

Over recent decades, China’s healthcare system has transitioned from a planned economic system to a market economic system, while also undergoing pressure to shift to a patient-centered medical model rather than a traditional one. Challenges arising from this transformation, including the uneven distribution of medical resources, subpar medical service quality, and rapidly rising medical costs, have affected patient satisfaction (Chen et al., 2021; Jakovljevic et al., 2023). Further, complaints about tense doctor–patient relationships and dissatisfaction with the quality of medical services have recently increased (Lv et al., 2021). Interestingly, patient satisfaction surveys conducted by various local governments and medical institutions have shown relatively high satisfaction levels (Sun et al., 2017). However, these surveys are often biased toward the perspectives of management or healthcare providers and may not fully reflect patients’ genuine opinions on medical service quality (Black et al., 2021). Moreover, they often fail to capture the complexity and breadth of medical services (Kingsley and Patel, 2017). Consequently, these satisfaction evaluations tend to be more positive than actual patient experiences, preventing the acquisition of valuable information that could be used to improve service quality (Bull et al., 2019; Bellio and Buccoliero, 2021). To some extent, this suggests a potential lack of understanding about patient satisfaction and the underlying psychological factors that influence it (Wang et al., 2023). Therefore, further research is necessary to gain comprehensive insights into patient satisfaction and the hidden psychological factors that influence it.

Research on patient satisfaction relies on traditional customer satisfaction theories that view patients as rational economic agents (Abu-Rumman et al., 2022). Common areas of focus include medical service quality, expenses, doctor–patient communication, and patient characteristics. However, this approach overlooks the impact of cognition, emotions, psychology, and the social environment of medical services on patient satisfaction, especially in the context of asymmetric medical service information. Bounded rationality, a critical theoretical framework in behavioral economics, suggests that people may not always make entirely rational decisions due to limitations in information acquisition, processing capability, and psychological biases (Jordão et al., 2020; Lejarraga and Pindard-Lejarraga, 2020). This means that patients may lack the comprehensive information necessary to assess their satisfaction with medical services. Additionally, they might struggle to make entirely rational judgments when dealing with complex medical information, and psychological factors such as emotions and expectations may cause deviations from rationality (Kheirkhah et al., 2020; Russo et al., 2021). Therefore, patient satisfaction assessments could be significantly enhanced by understanding and evaluating patient satisfaction based on the theory of bounded rationality.

For this reason, the present study uses this theory to determine the psychological factors that influence patient satisfaction at both the individual and the societal levels. In this way, it re-examines and analyzes current issues in patient satisfaction research and applications. It also investigates the impact of factors such as medical risk perception, negative emotions, the social cognition of the medical environment, and social desirability bias on patient satisfaction as well as the interactions between these factors. Specifically, it examines how these factors influence patient satisfaction directly and indirectly as well as their moderating effects to integrate behavioral economics theory into patient satisfaction research. This study also provides specific strategies and measures that medical institutions can use to assess and improve patient satisfaction more effectively.

## Theory and hypotheses

### Bounded rationality theory

Traditional economics and management science studies assume complete rationality as a starting point. However, as decision-making environments become more complex and information becomes more abundant, people’s cognitive judgment capabilities demonstrate bounded rationality. The theory of bounded rationality was proposed by the renowned economist Simon (1979, 2000). According to his theory, human behavior is rationally bounded in complex environments with limited information resources. This limitation has two principal manifestations: first, the accuracy of decision-making assessments is restricted because of the contradiction between the vagueness of the objective external environment and the precision required for rational analysis; second, human cognitive abilities such as memory and information processing are limited, leading to the inevitable influence of emotions, feelings, desires, will, social psychology, and other non-rational factors on decision-making and management practices (Gigerenzer, 2020). Compared with completely rational behavior, bounded rational behavior is mainly observed in cognitive biases, decision biases, and social preferences (Ren and Huang, 2018).

Patients evaluate individualized medical services as social beings rather than as rational economic agents (Chen et al., 2018). This manifests in two primary ways at the individual level. First, patients often face cognitive deficits in assessing medical risks due to information asymmetry. Their understanding of disease pathogenesis, diagnosis, treatment measures, and medical risks is often limited because of asymmetric information between doctors and patients, leading to irrational judgments (Ren and Huang, 2018). Second, emotional factors further complicate the decision-making process. Negative emotions such as anxiety and depression, often triggered by the illness itself, can impair judgment (Jeon et al., 2021). From a societal perspective, two additional elements come into play. The first is the influence of the social cognition of the medical environment. Rising tensions in doctor–patient relationships, sometimes escalating to violence, have eroded trust not only in individual healthcare providers but also in the medical system as a whole (Li and Khan, 2022). The second factor is social desirability bias. Doctors serve as agents, making treatment decisions based on their professional knowledge, while patients act as principals, entrusting the doctor with their disease treatment (Tan et al., 2022). Consequently, patients might provide answers that align more with social expectations than their true feelings, aiming to create a positive impression on medical staff and concealing aspects of the service with which they are dissatisfied (Baumgartner et al., 2021). In conclusion, patients’ evaluations of healthcare services fall short of the ideal standards of rational decision-making, such as comprehensive knowledge and unbiased information. Thus, it is evident that patients generally operate within the confines of bounded rationality when making healthcare decisions.

### Relations between medical risk perception, negative emotions, and patient satisfaction

Medical risk refers to factors or events that may occur during the medical process that may affect patient health and safety. While not always leading to catastrophic consequences, these risks exist objectively and cannot be entirely controlled, predicted, or avoided (Hanoch et al., 2018). Medical risk perception, on the contrary, refers to a patient’s understanding and cognition of the potential risks, side effects, and adverse consequences during the medical process (Pietrzykowski and Smilowska, 2021). Ozdemir and Finkelstein (2018) found that patients with a high level of risk perception were more inclined to actively participate in medical decision-making, which increased their satisfaction with and trust in the treatment plan. Similarly, Sharkiya (2023) discovered that detailed discussions of treatment risks and benefits could enhance patient acceptance of and satisfaction with treatment plans. Moreover, Frosch et al. (2003) found that patients with hypertension, when well informed about the risks involved in decision-making, are more likely to follow medical advice, thereby leading to better long-term blood pressure control, improved health outcomes, and increased satisfaction. Based on these findings, we propose the following hypothesis:

*H1*: High levels of medical risk perception positively affect patient satisfaction.

There is an established connection between a patient’s level of medical risk perception and negative emotions such as anger, shame, disgust, guilt, and fear (Alzahrani, 2021). Recent studies have reinforced the concept that health risk uncertainty is a significant trigger of negative emotions. For instance, Godara et al. (2023) found that increases in intolerance of uncertainty were associated with higher levels of stress, depression, and anxiety, mediated by difficulties in emotion regulation. This highlights the impact of cognitive control and flexibility in mental health, particularly in crisis situations. Similarly, Morriss et al. (2022) revealed that uncertainty, especially under ambiguous conditions, is more likely to elicit negative emotions such as fear, anxiety, sadness, and frustration. This underscores the broad impact of uncertainty on emotional experiences and its complex interplay with emotional well-being. Based on this, we propose the following hypothesis:

*H2*: High levels of medical risk perception positively affect patients’ negative emotions.

Additionally, negative emotions can cause patients to doubt medical advice, affecting their trust in and satisfaction with the treatment process (Gupta et al., 2014; Lerner et al., 2015). When patients experience anxiety or depression, they may exhibit negative attitudes toward communicating with doctors (Baldwin and Spears, 2019). Negative emotions can also influence treatment outcome expectations, resulting in lower satisfaction with treatment effectiveness (Glattacker et al., 2022; Wang et al., 2023). Based on these findings, we propose the following hypothesis:

*H3*: High levels of negative emotions negatively impact patient satisfaction.

Combined with the path and direction of H2 and H3, we further propose the following research hypothesis:

*H4*: Negative emotions mediate the relationship between medical risk perception and patient satisfaction, exhibiting a suppressive effect.

Emotions can act as a “signal” or “heuristic,” affecting people’s perception and evaluation of risk (Schudy et al., 2020). When patients’ negative emotions are stoked, they may focus more on medical risks and overestimate them. In this situation, the impact of medical risk perception on patient satisfaction may be strengthened (Alzahrani, 2021), as patients may evaluate medical services more negatively. Conversely, if patients do not experience strong negative emotions, they may not overfocus on or overestimate medical risks, thereby weakening the impact of medical risk perception on patient satisfaction (Wang et al., 2023). Hence, we propose the following hypothesis:

*H5*: Negative emotions positively moderate the relationship between medical risk perception and patient satisfaction.

### Relations between the social cognition of the medical environment, social desirability bias, and patient satisfaction

The social cognition of the medical environment refers to an individual’s understanding and perception of various social aspects of the medical environment, including the accessibility and fairness of medical services and doctor–patient relationships (Banerjee and Sanyal, 2012; Zhou et al., 2021). The significance of fairness in medical services, which greatly affects patient satisfaction, has been highlighted by Liang et al. (2017) and Kyle et al. (2021). Moreover, Katzenellenbogen et al. (2013) emphasized the importance of accessible patient-centered medical services in enhancing patient satisfaction. Additionally, several studies have indicated a strong correlation between doctor–patient relationships and patient satisfaction (Huynh and Dicke-Bohmann, 2020). Based on these findings, we propose the following hypothesis:

*H6*: The social cognition of the medical environment positively influences patient satisfaction.

Another relevant concept is social desirability bias, which refers to the tendency of individuals to behave or evaluate in a manner that conforms to others’ expectations, as influenced by social expectations and the environment, to gain approval and avoid adverse consequences (Larson, 2019; Tan et al., 2022). In environments in which doctor–patient conflicts are prominent, patients may exhibit more social desirability bias in their satisfaction evaluations, providing positive feedback to counterbalance negative social perceptions of the medical environment (Burgess and Huston, 2013; Gupta et al., 2014). Therefore, we propose the following hypothesis:

*H7*: The social cognition of the medical environment negatively affects social desirability bias.

Additionally, resource exchange theory suggests that interactions involve the exchange of resources and that the receiver responds through behavioral feedback, creating an interaction process (Cropanzano et al., 2017); thus, in the context of medical services, patients may seek to establish good relationships with medical staff to receive better treatment, leading to higher satisfaction. Furthermore, Badejo et al. (2022) found that the presence of social desirability bias can skew patient satisfaction scores, preventing them from truly reflecting patients’ sentiments. This distortion compromises the scores’ reliability and validity. Based on this, we propose the following hypothesis:

*H8*: Social desirability bias positively influences patient satisfaction.

Combined with the path and direction of H7 and H8, we further propose the following hypothesis:

*H9*: Social desirability bias mediates the relationship between the social cognition of the medical environment and patient satisfaction, exhibiting a suppressive effect.

Finally, drawing on social influence theory (Kwon et al., 2021) and expectancy confirmation theory (Oh et al., 2022), we propose that social desirability bias plays a crucial role in the relationship between the social cognition of the medical environment and patient satisfaction. Both theories emphasize how social expectations and pressures as well as individual expectations shape behaviors and attitudes. Social influence theory describes how patients may inflate their satisfaction to foster a positive doctor–patient relationship, resulting in “satisfaction inflation” (Raza et al., 2020). Expectancy confirmation theory emphasizes that satisfaction depends on the gap between service expectations and actual experience (Alam et al., 2022). When social pressure regulates expectations, patient satisfaction evaluations may deviate. In situations with high social desirability bias, patients may adjust their satisfaction evaluations to gain social acceptance, thus weakening the impact of the social cognition of the medical environment on patient satisfaction. Thus, in situations in which there is low social desirability bias, satisfaction evaluations are more likely to reflect patients’ true feelings about the medical experience, amplifying the impact of the social cognition of the medical environment on patient satisfaction. Accordingly, we propose the following hypothesis:

*H10*: Social desirability bias negatively moderates the relationship between the social cognition of the medical environment and patient satisfaction.

**Figure 1:**
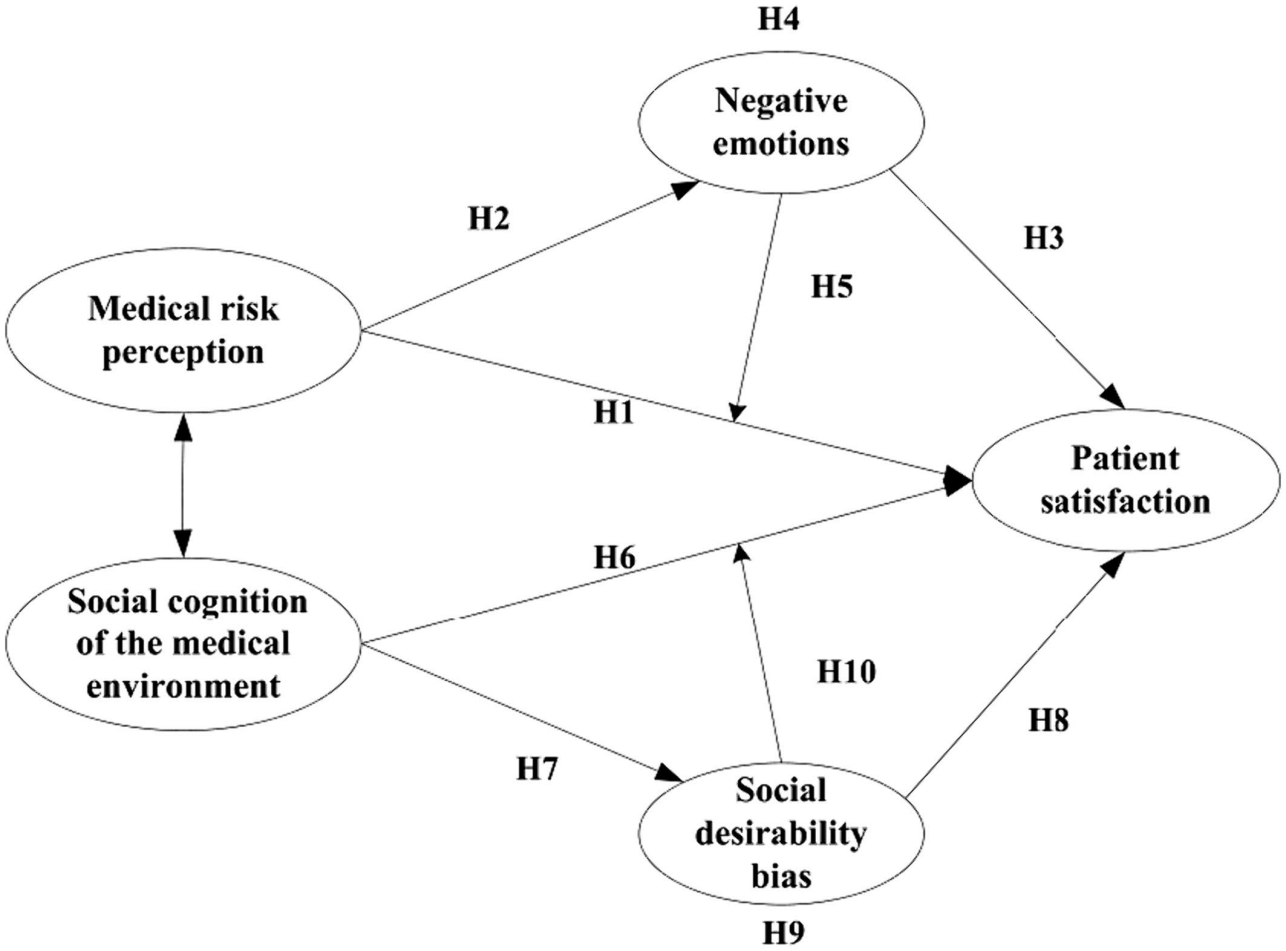
Proposed model.

In summary, there are correlations between medical risk perception, negative emotions, the social cognition of the medical environment, social desirability bias, and patient satisfaction. Negative emotions both mediate and moderate the relationship between medical risk perception and patient satisfaction. At the same time, social desirability bias mediates and moderates the relationship between the social cognition of the medical environment and patient satisfaction. The proposed relationships are illustrated in Figure 1.

## Materials and methods

### Participants and procedures

This study focused on hospitalized patients from 36 tertiary hospitals in Hangzhou, a thriving coastal city in southeastern China. We employed quota sampling and selected one tertiary hospital from each of the following eight districts of Hangzhou: Shangcheng, Gongshu, Xihu, Binjiang, Xiaoshan, Yuhang, Linping, and Qiantang. We surveyed 200 hospitalized patients in each selected hospital, resulting in a total sample size of 1,600 patients. According to our inclusion criteria, each patient: (1) was currently hospitalized or had just begun the discharge process, (2) was aged 18 years or older, (3) had been in hospital for at least 3 days, (4) provided informed consent and was willing to cooperate, and (5) was in stable condition and had the ability to independently express their feelings. We excluded patients who were (1) critically or terminally ill, (2) had mental or infectious diseases, and/or (3) explicitly declined to participate in the survey during their hospitalization. Patient recruitment and data collection for this study were conducted from September 1 to October 30, 2022. The common reasons for declining to participate in the study included concerns about privacy, a lack of interest, and feelings of fatigue or discomfort. From the 1,600 distributed questionnaires, we successfully collected 1,522, resulting in a high response rate of 95.13%. Questionnaires were deemed invalid if they had incomplete answers or if more than 50% of the questions had identical responses. After a thorough inspection, 1,442 questionnaires were considered valid, yielding an effective response rate of 94.74%.

Before carrying out the full-scale survey, a preliminary survey was conducted to agree an implementation plan and confirm the final version of the questionnaire. The formal survey was conducted by two researchers and five postgraduate students, all of whom had extensive field-survey experience. To ensure consistency, all investigators received uniform training on the survey standards and methods. The on-site investigation employed a one-on-one questionnaire completion method. Before the survey, informed consent was obtained from all participants and the necessary explanations were provided during the process. After completion, each questionnaire was thoroughly checked to ensure compliance with the requirements. The collected questionnaires were uniformly numbered and all data were entered twice to ensure accuracy.

### Measures

The sociodemographic information solicited included five items: sex (male, female), age (≤29, 30–39, 40–49, 50–59, ≥60), educational attainment (junior high school and below, high school, junior college, undergraduate or above), region of residence (local or non-local), and whether this was the patient’s first visit to hospital (yes, no). Participants’ demographic characteristics are shown in Table 1.

**Table 1:** Demographic characteristics of the participants.

Characteristic	Category	Frequency (*N*)	Composition ratio (%)
Sex	Male	677	46.9
	Female	765	53.1
Age (in years)	≤29	563	39.0
	30–39	355	24.6
	40–49	213	14.8
	50–59	134	9.3
	≥60	177	12.3
Educational attainment	Middle school or below	408	28.3
	High school	292	20.2
	Junior college	303	21.0
	Undergraduate or above	439	30.4
Region of residence	Local population	857	59.4
	Non-local population	585	40.6
First visit to hospital	Yes	435	30.2
	No	1,007	69.8

The reliability of the questionnaire was evaluated using internal consistency (Cronbach’s α coefficient) and composite reliability (CR), whereas the validity of the measurement was assessed through content and convergent validity. The questionnaire comprised several sections: sociodemographic information, a patient satisfaction scale, and questions evaluating medical risk perception, negative emotions, the social cognition of the medical environment, and social desirability bias (Table 2).

**Table 2:** Measurement items and results of the reliability and validity analysis of the questionnaire (*N* = 1,042).

Construct	Dimension	Item no.	Measurement item	Cronbach’s *α*	Factor loading	Correlation coefficient	AVE	CR
Patient satisfaction	Service environment (SEN)	SEN1	The hospital provides convenient and complete facilities.	0.857	0.680	0.786^**^	0.603	0.858
		SEN2	Clear and definite medical guidance instructions are available on each floor of the hospital.		0.743	0.835^**^		
		SEN3	The hospital waiting area is clean and comfortable.		0.843	0.870^**^		
		SEN4	The hospital outpatient examination room is clean and comfortable.		0.829	0.854^**^		
	Service efficiency (SE)	SE1	The waiting time for registration is reasonable.	0.846	0.735	0.827^**^	0.580	0.846
		SE2	The waiting time for examination and treatment is reasonable.		0.739	0.829^**^		
		SE3	The waiting time for the results of the inspection report is reasonable.		0.744	0.812^**^		
		SE4	The entire process, including registration, payment, and inspection, is convenient for you.		0.825	0.842^**^		
	Service attitude (SA)	SA1	The medical staff communicates with you in a polite and gentle tone.	0.856	0.717	0.808^**^	0.602	0.858
		SA2	The doctor patiently listens to you about your current condition and symptoms.		0.791	0.854^**^		
		SA3	The doctor thoroughly explains the specific treatment plan for your disease.		0.821	0.858^**^		
		SA4	The medical staff provides you with detailed instructions on drug use and precautions.		0.772	0.826^**^		
	Service technology (ST)	ST1	The doctor quickly diagnoses and identifies your symptoms or diseases.	0.838	0.762	0.826^**^	0.564	0.838
		ST2	The medical staff demonstrates skillful diagnosis and nursing operations.		0.762	0.824^**^		
		ST3	The doctor recommends undergoing necessary and reasonable examinations.		0.738	0.819^**^		
		ST4	The doctor provides targeted preventive health measures or recommendations for your condition.		0.743	0.813^**^		
	Medical expenses (ME)	ME1	You find the cost of outpatient consultation (treatment) reasonable.	0.879	0.816	0.861^**^	0.648	0.880
		ME2	You find the cost of the examination necessary and reasonable.		0.841	0.876^**^		
		ME3	You find the cost of medicine necessary and reasonable.		0.818	0.868^**^		
		ME4	The hospital’s medical fee collection standards are open, transparent, and easy to inquire about.		0.741	0.821^**^		
Psychological factors based on bounded rationality	Medical risk perception (MRP)	MRP1	There are potential medical risks in the treatment process.	0.825	0.726	0.796^**^	0.545	0.827
		MRP2	Medical risks exist throughout the diagnosis and treatment process.		0.804	0.833^**^		
		MRP3	All drugs have potential adverse reactions.		0.727	0.815^**^		
		MRP4	Doctors are involved in high-risk work.		0.691	0.799^**^		
	Negative emotions (NE)	NE1	You may feel worried.	0.885	0.794	0.852^**^	0.659	0.885
		NE2	You may feel nervous.		0.850	0.879^**^		
		NE3	You may feel annoyed.		0.826	0.868^**^		
		NE4	You may feel sad.		0.776	0.846^**^		
	Social cognition of the medical environment (SCME)	SCME1	What is your perception of the current doctor–patient relationship in China?	0.750	0.684	0.809^**^	0.502	0.751
		SCME2	How difficult is it for you to see a doctor in the current social medical environment?		0.752	0.824^**^		
		SCME3	What is your perception of the fairness of healthcare services among different income groups?		0.688	0.817^**^		
	Social desirability bias (SDB)	SDB1	In your opinion, will the quality of follow-up medical services you receive be affected by your evaluation of medical staff?	0.757	0.725	0.824^**^	0.510	0.757
		SDB2	Even if you are dissatisfied, you will try to avoid expressing it publicly or use euphemistic words as much as possible?		0.717	0.819^**^		
		SDB3	If there are no specific unsatisfactory aspects during hospitalization, the satisfaction evaluation will receive high marks if it deserves them?		0.699	0.818^**^		

CR, composite reliability; AVE, average variance extracted. ^**^p < 0.01, two-tailed test.

The Patient Satisfaction Scale was adapted from the Inpatient Satisfaction Evaluation Scale developed by Chen et al. (2018), with individual item adjustments. The final satisfaction scale consisted of five dimensions: service environment, service efficiency, service attitude, service technology, and service cost. In this study, the Cronbach’s *α* for the Patient Satisfaction Scale was 0.964. Each dimension had a Cronbach’s *α* between 0.838 and 0.879, a CR between 0.838 and 0.880, and an average variance extracted (AVE) between 0.564 and 0.648. These values indicated the measures had good reliability and validity (Table 2).

The Medical Risk Perception Scale was based on the Lung Cancer Patient Risk Perception Measurement Questionnaire developed by Park et al. (2009). It included four items, each rated on a 5-point Likert scale (1 = strongly disagree to 5 = strongly agree). For the Medical Risk Perception Scale, the Cronbach’s α was 0.825, the CR value was 0.827, the factor loading ranged from 0.691 to 0.804, the AVE value was 0.545, and the correlation coefficient between each item and the overall score was 0.796–0.833 (*p* < 0.001), indicating the good reliability and validity of the measures (Table 2).

The Negative Emotion Scale in the patient questionnaire was adapted from the Positive and Negative Emotion Scale compiled by Watson et al. (1988), selecting worry, tension, irritability, and sadness as the four adjectives to describe negative emotions. A 5-point Likert scale was used for scoring (1 = very mild or none to 5 = very strong), and respondents were asked to rate the intensity of each negative emotion they experienced during their hospital stay. For the Negative Emotion Scale, the Cronbach’s *α* was 0.885, the CR value was 0.885, factor loading ranged from 0.776 to 0.850, the AVE value was 0.659, and the correlation coefficient between each item and the overall score was 0.846–0.879 (*p <* 0.001), indicating the good reliability and validity of the measures (Table 2).

The Social Cognition of the Medical Environment Scale developed by our research team primarily reflected patients’ understanding and perception of the accessibility, fairness, and doctor–patient relationship in the current social environment. The scale consisted of three items. The first item asked “How do you perceive the current doctor–patient relationship in our country?” Responses were scored on a 5-point Likert scale (1 = very tense to 5 = very harmonious). The second item asked “How do you feel about seeking medical services in the current social medical environment?” Responses were scored on a 5-point Likert scale (1 = very difficult to 5 = very easy). Lastly, the third item asked “What is your perception of the fairness of healthcare services among different income groups?” Responses were scored on a 5-point Likert scale (1 = very unfair to 5 = very fair). For the Social Cognition of the Medical Environment Scale, the Cronbach’s *α* was 0.750, the CR value was 0.751, factor loading ranged from 0.684 to 0.752, the AVE value was 0.502, and the correlation coefficient between each item and the overall score was 0.809–0.824 (*p <* 0.001), indicating the good reliability and validity of the measures (Table 2).

The Social Desirability Bias Scale was also developed by our research team, drawing on the insights from Uziel (2010) and Perinelli and Gremigni (2016). These studies suggested that social desirability bias is most evident in domains such as psychological defense, impression management, and interpersonal adaptation. Based on these theoretical frameworks, we created three questionnaire items to assess this bias among patients. The first item asked “In your opinion, will the quality of follow-up medical services you receive be affected by your evaluation of medical staff?” Responses were scored on a 5-point Likert scale (1 = strongly disagree to 5 = strongly agree). The second item asked “Even if you are dissatisfied, you will try to avoid expressing it publicly or use euphemistic words as much as possible?” Responses were scored on a 5-point Likert scale (1 = strongly disagree to 5 = strongly agree). Lastly, the third item asked “If there are no specific unsatisfactory aspects during hospitalization, the satisfaction evaluation will receive high marks if it deserves them?” Responses were scored on a 5-point Likert scale (1 = strongly disagree to 5 = strongly agree). The Cronbach’s α for the Social Desirability Bias Scale was 0.757, the CR value was 0.757, factor loading ranged from 0.699 to 0.725, the AVE value was 0.510, and the correlation coefficient between each item and the overall score was 0.818–0.824 (*p* < 0.001), indicating the good reliability and validity of the measures (Table 2).

### Statistical analysis

Statistical analysis was conducted using SPSS (version 26.0) and AMOS (version 24.0) (IBM Corporation, Armonk, NY, USA). Data were analyzed using descriptive statistics such as frequency and constituent ratio. The differences in patient satisfaction scores by demographic characteristics were analyzed using a *t*-test and one-way ANOVA. Pearson’s correlation analysis was used to calculate the correlations between medical risk perception, the social cognition of the medical environment, negative emotions, social desirability bias, patient satisfaction, and their dimensions. Hierarchical multiple regression analysis (using the enter method) was employed to analyze the main factors influencing patient satisfaction. A forward stepwise multiple regression analysis using patient satisfaction as a dependent variable was also applied. All variables that may have influenced patient satisfaction were added into the model to mitigate any possible confounding. Structural equation modeling was employed to explore the mechanisms by which medical risk perception, the social cognition of the medical environment, negative emotions, and social desirability bias influence patient satisfaction and to calculate the corresponding effect sizes. The bootstrap method validated the mediating roles of negative emotions and social desirability bias (MacKinnon, 2012). Finally, the two-step technique proposed by Ping (1995) was used to analyze the moderating effects of negative emotions and social desirability bias. The resulting model incorporated two paths: medical risk perception → negative emotions → patient satisfaction and the social cognition of the medical environment → social desirability bias → patient satisfaction. The analysis results demonstrated the model’s strong fit with the sample data.

## Results

### Patient satisfaction by demographic characteristics

The results showed significant differences (*p* < 0.05) in patients’ satisfaction scores based on their age, educational attainment, region of residence, and whether it was their first visit to hospital (Table 3).

**Table 3:** One-way ANOVA of patient satisfaction with different demographic characteristics.

Characteristic	Category	Frequency (*N*)	Patient Satisfaction	*F*-value	*p*-value
Age (in years)	<29	563	74.20 ± 10.32	3.202	0.013
	30–39	355	72.63 ± 10.49		
	40–49	213	71.60 ± 10.34		
	50–59	134	72.98 ± 10.79		
	≥60	177	72.11 ± 11.15		
Educational attainment	Middle school or below	408	72.53 ± 11.08	6.131	<0.01
	High school	292	71.89 ± 9.98		
	Junior college	303	72.35 ± 10.09		
	Undergraduate or above	439	74.82 ± 10.53		
Region of residence	Local	857	72.10 ± 10.57	17.780	<0.01
	Non-local	585	74.47 ± 10.35		
First visit to hospital	Yes	435	74.70 ± 10.28	15.132	<0.01
	No	1,007	72.35 ± 10.58		

### Descriptive statistics and correlation analyses

The results of the descriptive statistics and correlation analyses are presented in Table 4. Patient satisfaction was significantly correlated with medical risk perception (*r* = 0.313, *p* < 0.01), the social cognition of the medical environment (*r* = 0.380, *p* < 0.01), negative emotions (*r* = −0.259, *p* < 0.01), and social desirability bias (*r* = 0.286, *p* < 0.01). Furthermore, medical risk perception was significantly correlated with negative emotions (*r* = 0.179, *p* < 0.01), and the social cognition of the medical environment was significantly correlated with social desirability bias (*r* = −0.174, *p* < 0.01). Additionally, a significant correlation was observed between medical risk perception and the social cognition in the medical environment (*r* = 0.179, *p* < 0.01).

**Table 4:** Descriptive statistics and correlation analyses.

		**Mean**	**Standard deviation**	**1**	**2**	**3**	**4**	**5**
1	MRP	3.698	0.696	1				
2	NE	2.126	0.897	0.179**	1			
3	SCME	2.976	0.709	0.179**	0.012	1		
4	SDB	3.231	0.793	0.112**	−0.112**	−0.174**	1	
5	PS	3.653	0.527	0.313**	−0.259**	0.380**	0.286**	1

^**^p < 0.01, two-tailed test. For all variables, possible scores ranged from 1 to 5.

### Hierarchical multiple regression analysis of patient satisfaction

In the one-way ANOVA of patient satisfaction, the dummy variables (sex, age, educational attainment, region of residence, and whether it was the patient’s first visit) represented unordered categorical data with statistical significance. Subsequently, a hierarchical multiple regression analysis was conducted with patient satisfaction as the dependent variable. The five models used were as follows: (1) demographic characteristics; (2) demographic characteristics + medical risk perception; (3) demographic characteristics + medical risk perception + negative emotions; (4) demographic characteristics + medical risk perception + negative emotions + the social cognition of the medical environment; and (5) demographic characteristics + medical risk perception + negative emotions + the social cognition of the medical environment + social desirability bias.

The results demonstrated that when demographic characteristics, medical risk perception, negative emotions, the social cognition of the medical environment, and social desirability bias were included in the regression equation, the change in *R*^2^ (Δ*R*^2^) was statistically significant. This showed that the respective impacts of medical risk perception, negative emotions, the social cognition of the medical environment, and social desirability bias on patient satisfaction were all stronger than that of demographic characteristics. Among these factors, the impact of the social cognition of the medical environment was the most substantial, accounting for 10.3% of the variance in patient satisfaction.

Furthermore, the analysis results for the fifth model (Table 5) indicated that non-local patients scored higher than local patients and that patients who were experiencing their first hospital visit scored higher than those who were not. Patient satisfaction also increased with higher scores for medical risk perception, the social cognition of the medical environment, and social desirability bias (*β* = 0.253, *p* < 0.01; *β* = 0.387, *p* < 0.01; *β* = 0.287, *p* < 0.01) and decreased with higher negative emotion scores (*β* = −0.269, *p* < 0.01).

**Table 5:** Hierarchical multiple regression analysis of patient satisfaction.

Variable	First block	Second block	Third block	Fourth block	Fifth block
	Standardized beta	Standardized beta	Standardized beta	Standardized beta	Standardized beta
Age (in years) (≤29 = reference group)					
30–39	−0.035	−0.036	−0.004	−0.003	0.002
40–49	−0.046	−0.037	−0.026	−0.020	−0.009
50–59	0.013	0.013	0.032	0.038	0.038
≥60	−0.011	−0.014	0.012	0.018	0.032
Educational attainment (middle school or below = reference group)					
High school	−0.011	−0.030	−0.039	−0.024	−0.010
Junior college	−0.002	−0.008	−0.030	−0.013	−0.005
Undergraduate or above	0.100^**^	0.059	0.017	0.048	0.052
Region of residence (Local population = reference group)					
Non-local population	0.094^**^	0.091^**^	0.059^*^	0.052^*^	0.052^*^
First visit to hospital (Yes = reference group)					
No	−0.076^**^	−0.068^**^	−0.067^**^	−0.067^**^	−0.054^**^
MRP		0.302^**^	0.364^**^	0.302^**^	0.253^**^
NE			−0.316^**^	−0.307^**^	−0.269^**^
SCME				0.328^**^	0.387^**^
SDB					0.287^**^
*R^2^*	0.034	0.124	0.215	0.319	0.395
*F*	5.552^**^	20.185^**^	35.709^**^	55.745^**^	71.667^**^
△*R^2^*	0.034	0.090	0.092	0.103	0.076
△*F*	5.552^**^	146.793^**^	167.469^**^	216.847^**^	179.280^**^
VIFmax	2.034	2.053	2.073	2.082	2.082

VIFmax, maximum variance inflation factor. *p < 0.05; **p < 0.01.

### Model construction

The structural equation model included medical risk perception and the social cognition of the medical environment as latent independent variables, patient satisfaction as a latent dependent variable, and negative emotions and social desirability bias as latent mediator variables. The structural model demonstrated a satisfactory fit (χ^2^ = 399.770, df = 145, *p* < 0.001, χ2/df = 2.757 < 3, GFI = 0.971 > 0.9, AGFI = 0.963 > 0.9, SRMR = 0.045 < 0.05, RMSEA = 0.035 < 0.05, CFI = 0.977 > 0.9, TLI = 0.973 > 0.9, NFI = 0.964 > 0.9). The overall model fit index met the recommended values (Hu and Bentler, 1999; Hair et al., 2010), indicating a good fit for the partial model of the influencing mechanism of patient satisfaction based on the psychological factors of bounded rational behavior.

### Path analysis

Table 6 presents the results of the analysis of the standardized effects. First, medical risk perception positively impacted patient satisfaction, with a standardized path coefficient of 0.302 (*p* < 0.01), supporting H1. Second, negative emotions negatively impacted patient satisfaction, with a standardized path coefficient of −0.335 (*p* < 0.01), supporting H2. Additionally, medical risk perception had a positive impact on negative emotions, with a standardized path coefficient of 0.202 (*p* < 0.01), supporting H3. Furthermore, the social cognition of the medical environment positively impacted patient satisfaction, with a standardized path coefficient of 0.511 (*p* < 0.01), supporting H6. Moreover, social desirability bias positively impacted patient satisfaction, with a standardized path coefficient of 0.405 (*p* < 0.01), supporting H7. Lastly, the social cognition of the medical environment negatively impacted social desirability bias, with a standardized path coefficient of −0.218 (*p* < 0.01), supporting H8.

**Table 6:** Results of the hypothesis testing and mediating effects analysis.

Relation between the variables	Standardized direct effect	S.E.	Bias-corrected 95%CI			Percentile 95%CI			Supported hypotheses
			Lower	Upper	*p*	Lower	Upper	*p*	
MRP → PS	0.302	0.030	0.242	0.359	0.001	0.244	0.361	0.001	H1
NE → PS	−0.335	0.024	−0.382	−0.288	0.001	−0.384	−0.289	0.001	H2
MRP → NE	0.202	0.030	0.144	0.260	0.001	0.142	0.259	0.001	H3
SCME→PS	0.511	0.029	0.455	0.568	0.001	0.453	0.565	0.001	H6
SDB → PS	0.405	0.030	0.349	0.463	0.001	0.347	0.462	0.001	H7
SCME→SDB	−0.218	0.038	−0.293	−0.144	0.001	−0.290	−0.140	0.001	H8

### Mediating effect

The total effects of medical risk perception and the social cognition of the medical environment on patient satisfaction were 0.235 and 0.423, respectively. Neither the bias-corrected 95%CI nor the percentile 95%CI included a 0, indicating a significant overall mediating effect. Further analysis revealed that the direct effects of medical risk perception and the social cognition of the medical environment on patient satisfaction were 0.302 and 0.511, respectively. The indirect effects were −0.068 and −0.088, respectively. Again, neither the bias-corrected 95%CI nor the percentile 95%CI of these effects included a 0. These results confirmed that both medical risk perception and the social cognition of the medical environment had significant direct and indirect effects on patient satisfaction, with a partial mediating effect, thus supporting H4 and H9 (Table 7).

**Table 7:** Examination of the mediating effects with the bootstrap method (standardized coefficients).

Path	Effect of type	Effect size	S.E.	Bias-corrected 95%CI			Percentile 95%CI			Supported hypotheses
				Lower	Upper	*p*	Lower	Upper	*p*	
MRP → PS	Total effects	0.235	0.032	0.17	0.298	0.001	0.170	0.297	0.001	
	Direct effects	0.302	0.030	0.242	0.359	0.001	0.244	0.361	0.001	
	Indirect effects	−0.068	0.012	−0.094	−0.046	0.001	−0.094	−0.045	0.001	H4
SCME→PS	Total effects	0.423	0.029	0.365	0.480	0.001	0.363	0.478	0.001	
	Direct effects	0.511	0.029	0.455	0.568	0.001	0.453	0.565	0.001	
	Indirect effects	−0.088	0.019	−0.129	−0.054	0.001	−0.127	−0.053	0.001	H9

### Moderating effects

**Figure 2:**
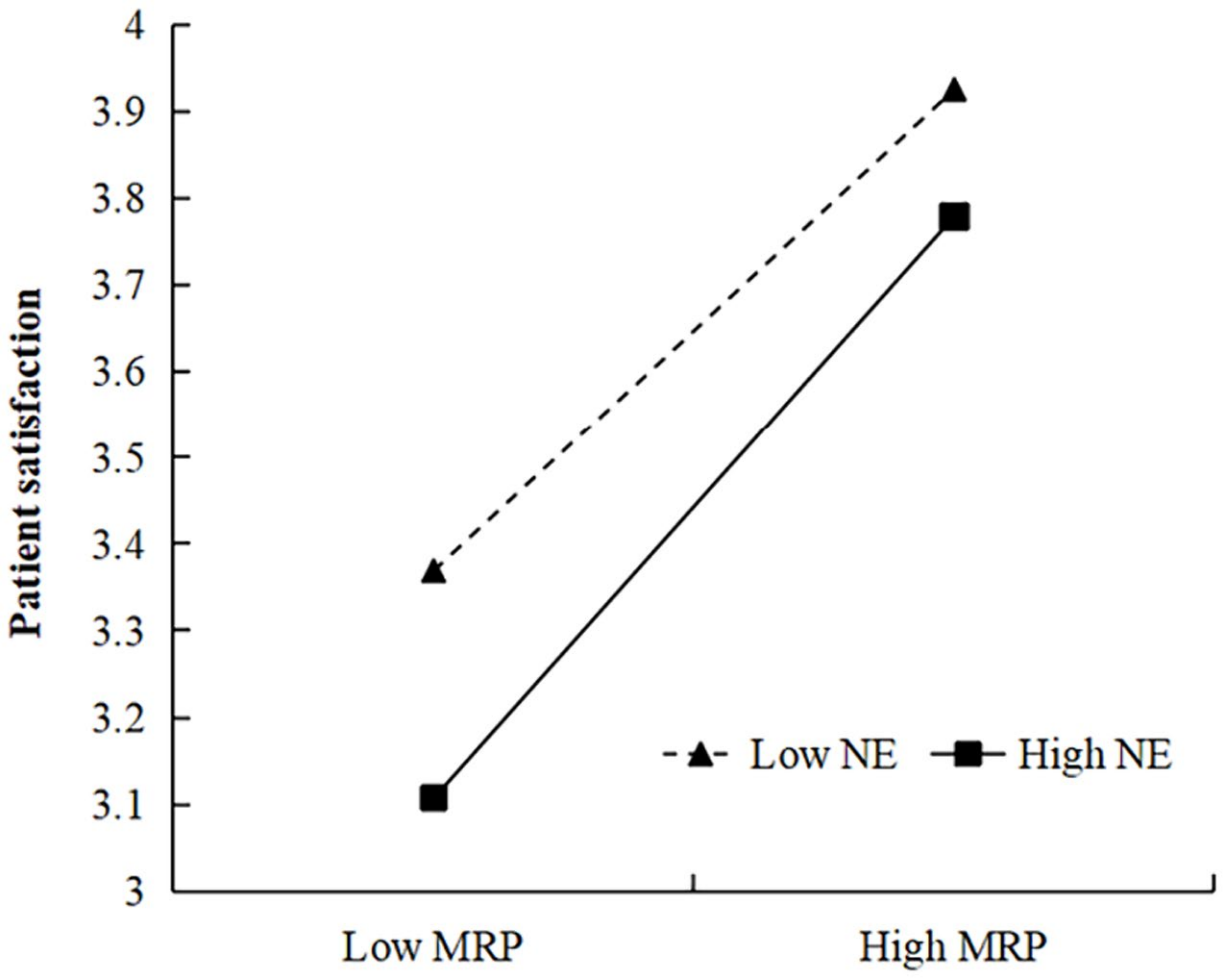
Moderating effect of negative emotions (NE) in the relation between medical risk perception (MRP) and patient satisfaction.

**Figure 3:**
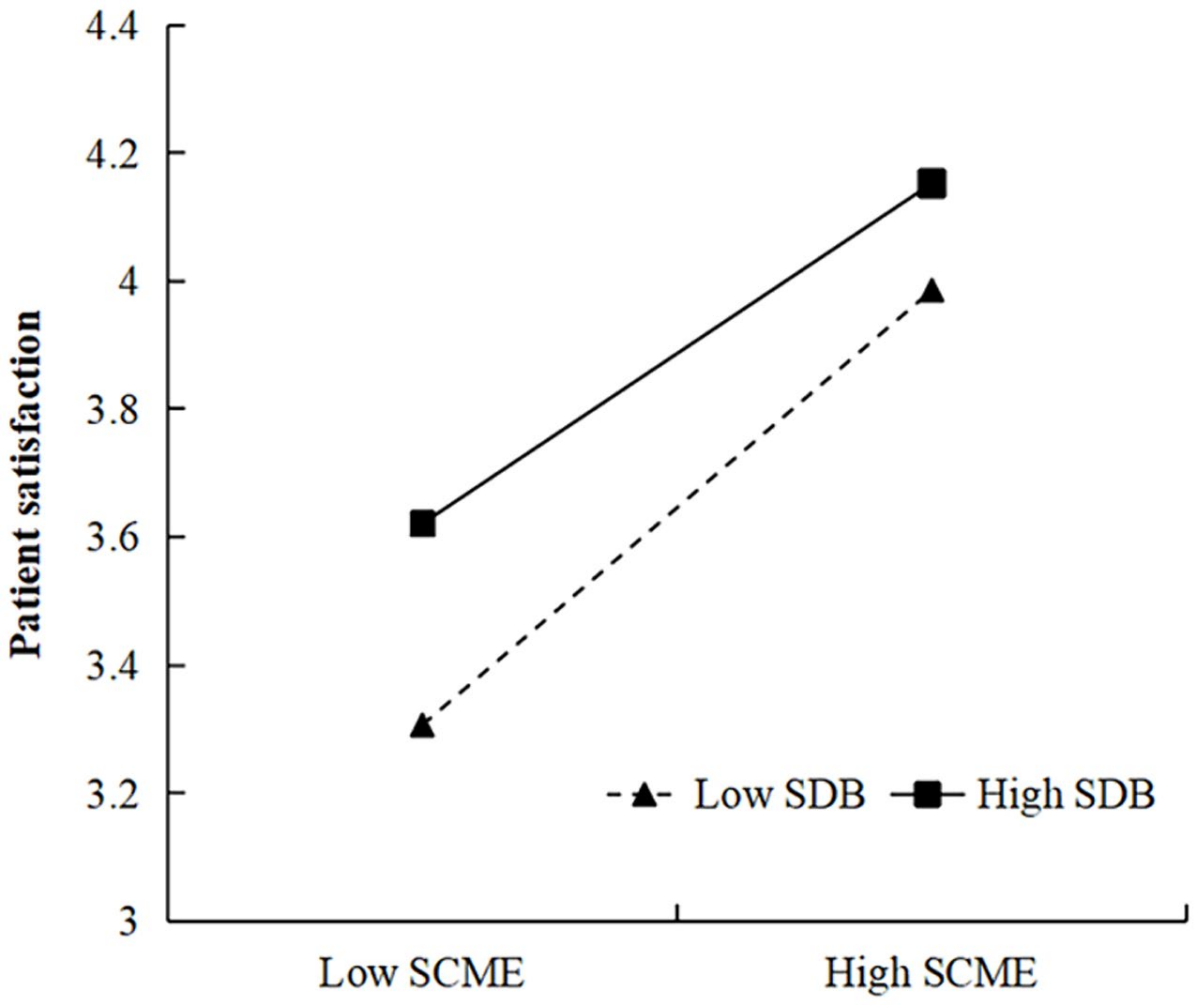
Moderating effect of social desirability bias (SDB) in the relation between the social cognition of the medical environment (SCME) and patient satisfaction.

Our results demonstrated significant moderating effects in both the negative emotions → patient satisfaction and social desirability bias → patient satisfaction paths. In the former, the influence of medical risk perception was significantly positive (*β* = 0.100, *p* < 0.01), supporting H5. Conversely, the latter path showed that the social cognition of the medical environment had a significantly negative impact (*β* = −0.150, *p* < 0.01), supporting H10 (Table 8). To delve deeper into the moderating effects of medical risk perception and the social cognition of the medical environment, we used a simple slope test to study the interaction effect model. By incorporating the mean value plus or minus the standard deviation of both the independent variable and the moderator into the regression equation, we generated a straightforward effect graph, depicted in Figure 2. Notably, the influence of medical risk perception on patient satisfaction was accentuated when patients experienced more negative emotions. Similarly, as shown in Figure 3, patient satisfaction increased with higher social desirability bias. However, when social desirability bias was high, the positive impact of the social cognition of the medical environment on patient satisfaction tended to decrease.

**Table 8:** Examination of the moderating effects.

Relations between the variables	Unstd.	Std.	Standard errors	*p*	Supported hypothesis
MRP*NE → PS	0.077	0.100	0.025	0.001	H5
SCME*SDB → PS	−0.124	−0.150	0.028	0.001	H10

## Discussion

This study, grounded in bounded rationality theory, delved into the psychological determinants of patient satisfaction, unveiling their complex interplay. Our findings align with those in the existing literature (Ai et al., 2022; Ali et al., 2023), underscoring the intricate dynamics at both the individual and the societal levels. At the individual level, we discovered that patient satisfaction is shaped by their medical risk perception and the influence of negative emotions. These negative emotions serve dual roles, acting as both mediators and positive moderators in the relationship between medical risk perception and patient satisfaction. At the societal level, social desirability bias is identified as a pivotal factor, functioning as a mediator and negative moderator between the social cognition of the medical environment and patient satisfaction. These insights align with those of prior studies (Xesfingi and Vozikis, 2016; Wang et al., 2023), emphasizing the nuanced nature of patient satisfaction that extends beyond traditional measures of service quality. They highlight the criticality of considering cognitive processes, emotional states, and social influences for a holistic understanding of patient satisfaction.

From an individual perspective, this study revealed significant relationships between medical risk perception, negative emotions, and patient satisfaction. Initially, we observed that medical risk perception positively influences patient satisfaction (H1), corroborating the findings of Jordão et al. (2020) and Sharkiya (2023). This link can be attributed to the alignment between patients’ expectations of treatment outcomes and actual results. A clear understanding of medical risks leads to more realistic outcome expectations, thereby narrowing the gap between expected and actual treatment effects and enhancing satisfaction, as supported by Parasuraman et al. (2018). This suggests that medical service providers should focus not only on communicating risks effectively but also on ensuring that patients’ expectations align closely with potential outcomes.

Furthermore, a notable correlation was found between medical risk perception and negative emotions, indicating that a heightened medical risk perception may induce negative emotions (H3), which in turn adversely affects patient satisfaction (H2). This relationship underscores the indirect influence of medical risk perception on patient satisfaction through the channel of negative emotions (H4), which is similar to the view of Schenker et al. (2011). Risk perception theory (Yilma et al., 2022) suggests that risk perception can trigger negative emotional reactions in individuals (Anderson et al., 2019). These reactions are typically composed of triggering events, emotional experiences, and assessments (Clore and Ortony, 2013). Therefore, an individual’s emotional experiences shape their judgment and evaluation, and negative sentiments can result in reduced satisfaction across different aspects (Di Castro et al., 2018; Deng et al., 2023). In the context of healthcare services, negative emotions among patients can lead to outcomes such as dissatisfaction, decreased trust in physicians, and doubts regarding treatment efficacy (Mach et al., 2019).

The mediating effects analysis revealed that the direct and indirect influences of medical risk perception on patient satisfaction moved in opposite directions, indicating a suppressive effect of negative emotions on this relationship (Zhu et al., 2023). This suggests that anxiety and fear can distort patients’ perception and processing of medical information, shifting focus to more negative aspects and, consequently, impacting their overall satisfaction. Moreover, the interaction between medical risk perception and negative emotions is complex, influenced by factors such as psychological resilience and specific coping strategies such as stress management techniques and therapeutic interventions (Vella and Pai, 2019; Troy et al., 2023). This complexity highlights the need for healthcare providers to incorporate psychological support, including counseling and resilience-building programs, as integral components of patient care.

In addition, this study shed light on the moderating role of negative emotions in the medical risk perception–negative emotions–patient satisfaction pathway (H5). This finding is similar to those of Gan and Fu (2022) and Meng et al. (2023). The results showed that a high degree of negative emotions can enhance patients’ sensitivity to medical risks and magnify their impact on satisfaction. Therefore, when patients experience severe negative emotions, they may show a heightened sensitivity toward medical risks, thereby escalating the influence of this factor on their satisfaction. By contrast, when patients’ negative emotions are less intense, they could demonstrate more tolerance and understanding of the inherent risks of the medical process, resulting in increased satisfaction with treatment outcomes. This suggests that patients with a high degree of medical risk perception might exhibit enhanced rational judgment in medical scenarios, equipping them to better manage the effect of negative emotions and consequently lowering the impact of such emotions on satisfaction. This can be accomplished by supplying more extensive information to help patients comprehend and accept medical risks as well as by fostering a more congenial and inviting medical environment to alleviate negative emotions.

From a macrosocial perspective, this study established significant connections between the social cognition of the medical environment, social desirability bias, and patient satisfaction. We found support for the hypothesis that a positive relationship exists between the social cognition of the medical environment and patient satisfaction (H6), as found by Liu (2015) and Chandra et al. (2018). Specifically, this relationship is rooted in how patients perceive the accessibility of healthcare services, fairness of healthcare delivery, and quality of interactions between doctors and patients. These perceptions, in turn, influence the overall satisfaction with healthcare services, echoing the findings of Ai et al. (2022) and Wang et al. (2023). To improve the public perception of healthcare fairness and accessibility, the government and relevant health agencies should focus on three key areas. First, they should bolster the service capabilities of primary healthcare institutions. Second, they should broaden both the scope and the depth of medical insurance coverage. Lastly, they should use the media to disseminate positive narratives about the healthcare environment. By implementing these targeted strategies, we can expect a subsequent rise in patient satisfaction.

This study also offered key insights into the role of social desirability bias in shaping patient satisfaction. It revealed that social desirability bias is positively correlated with patient satisfaction (H7), while the social cognition of the medical environment has a negative effect on this bias (H8). Moreover, the study found that the social cognition of the medical environment indirectly influences patient satisfaction through its impact on social desirability bias (H9). Rather than merely comprising a collective mindset, the social cognition of the medical environment encompasses society’s broad perceptions, emotions, and attitudes toward the doctor–patient relationship. When this cognition skews toward negativity or indifference, it discourages patients from providing honest satisfaction ratings. This reluctance stems from the fear that low scores could lead to dissatisfaction among medical staff, which could, in turn, compromise the quality of future medical care (Dunsch et al., 2018).

Patient satisfaction is particularly vulnerable to social psychological factors because of the inherent information asymmetry, unequal power dynamics, and frequent interactions between doctors and patients, which make it distinct from traditional customer satisfaction metrics (Croker et al., 2013; Xesfingi and Vozikis, 2016). Our mediating effects analysis indicated that the direct and indirect effects of the social cognition of the medical environment on patient satisfaction are contradictory, likely due to the suppressive effect of social desirability bias on this relationship (Cui et al., 2022). To address these complexities, it is essential to acknowledge the influence of social desirability bias when evaluating patient satisfaction. Effective countermeasures could include the implementation of a comprehensive patient education program comprising multimedia educational materials and online courses aimed at empowering patients to more accurately assess their healthcare experiences. Additionally, healthcare providers should consider hosting regular seminars to facilitate open dialogue between doctors and patients as well as implementing anonymous feedback systems to encourage honest and constructive communication.

Finally, this study discovered a negative moderating effect of social desirability bias in the social cognition of the medical environment → social desirability bias → patient satisfaction pathway (H10). This implies that when social desirability bias is prevalent, the influence of the social cognition of the medical environment on patient satisfaction is lessened. This supports the theory that individuals’ attitudes and behaviors are shaped by the social environment and its norms (Ajzen, 2020). Social desirability bias might cause patients to base their evaluations and reactions to the medical environment more on societal expectations and pressures than on their actual experiences and sentiments, thus reducing the influence of the social cognition of the medical environment on patient satisfaction. This discovery, which has been somewhat overlooked in existing patient satisfaction research, is a significant contribution to the field. Earlier empirical studies largely focused on factors such as the quality of medical services and doctor–patient relationships (Hesse and Rauscher, 2019; Bellio and Buccoliero, 2021), leaving a gap in the understanding of the impact of social psychological factors on patient satisfaction. The support of H10 not only underscores the importance of these factors in shaping patient satisfaction but also adds depth to theoretical research in this area.

### Theoretical implications

This study makes three theoretical contributions to the literature. First, it applies the theory of bounded rationality to patient satisfaction, offering a fresh perspective of patient behavior. Second, it uses empirical evidence to reveal the specific mechanisms through which patient satisfaction evaluations are affected by factors of bounded rationality such as medical risk perception, negative emotions, the social cognition of the medical environment, and social desirability bias. This enriched understanding of patient behavior provides practical guidance for improving patient satisfaction evaluation systems. Lastly, the study emphasizes the critical role of social psychological factors, particularly social desirability bias, in shaping patient satisfaction. This focus adds a new layer of complexity to existing models and offers healthcare providers and policymakers novel strategies for more precise evaluations and improvements in patient satisfaction. Furthermore, our theoretical contribution lies in demonstrating how bounded rationality theory should be applied to patient satisfaction evaluations, deepening our understanding of the theory and providing a new perspective of how to improve these evaluations.

### Practical implications

The practical contributions of this study can be grouped into three main categories. First, the findings provide a strategic basis for improving services offered by medical institutions. To address medical risk perception, institutions should improve risk education and communication to help patients better understand medical risks and reduce unnecessary fear and misunderstanding. Additionally, offering psychological counseling services can alleviate negative emotions such as anxiety and stress, leading to improved mental states for patients. Furthermore, implementing an anonymous, patient-centered evaluation and feedback system can mitigate social desirability bias to provide an objective and fair evaluation mechanism.

Second, the research results offer valuable references for governments and the relevant health departments to formulate policies and regulations. Governments can create policies that help medical institutions enhance service quality, expand medical insurance coverage and protection, improve the capacity of primary medical institutions, and promote positive media publicity. These steps could improve the public’s social cognition of the medical environment, fostering greater acceptance and trust.

Finally, the study’s findings provide a reference for the establishment of a scientific patient satisfaction evaluation model. The complexity of evaluating patient satisfaction arises not only from the diversity of the necessary indicators, such as the quality of medical services and doctor–patient communication, but also from the varying roles of the evaluating entities, be they government health departments, hospitals, or third-party organizations. These entities may influence patients’ cognitive and social desirability biases. Therefore, it is strongly recommended that the health departments of the Chinese government establish a multidisciplinary, multidimensional third-party evaluation organization. This body should comprise representatives from higher education institutions, specialized social survey organizations, and the media to ensure that evaluation indicators are selected and weighted in a manner that is both fair and transparent, thereby enhancing the reliability and validity of assessments.

### Limitations and future studies

This study has several limitations. First, as a cross-sectional study, we could only establish correlations, rather than causal relationships. Moreover, the sample data were limited to 1,442 hospitalized patients in Hangzhou, which may not fully represent patient satisfaction in other regions or types of hospitals. Thus, further studies that expand the survey scope and increase the sample size are necessary to enhance the generalizability of the results. Future studies should consider expanding the survey to a nationwide scale or including various types of medical institutions.

Second, the use of self-reporting in the questionnaire survey may have introduced common method bias (Baumgartner et al., 2021). To address this, future research should verify and analyze the findings using objective data such as actual medical service conditions as well as in-depth interviews.

Third, while this study explores the influencing mechanisms of four bounded rationality factors on patient satisfaction, it does not consider other potential factors such as overoptimism bias or stereotypes. Future research should incorporate these factors to gain a more comprehensive understanding of patient satisfaction. Social experiments in controlled environments may also help more accurately measure the impact of bounded rationality factors on patient satisfaction, providing deeper insights into their mechanisms.

## Conclusion

The results of this study indicated that hospitalized patients in Hangzhou had a medium level of overall satisfaction. Factors such as medical risk perception, negative emotions, the social cognition of the medical environment, and social desirability bias significantly influenced patient satisfaction. These findings reveal the impact of bounded rationality factors on the patient satisfaction evaluation process, highlighting the role of cognition, emotions, and social expectations. Specifically, patients’ negative emotions act as a masking mediator in the medical risk perception → patient satisfaction path and have a positive moderating effect. Similarly, social desirability bias plays a mediating role in the social cognition of the medical environment → patient satisfaction path, with a suppressive effect and a negative moderating effect. These insights offer a new perspective of patient satisfaction and valuable guidance for medical practice. Medical institutions should take proactive measures to manage patients’ negative emotions, communicate accurate medical risk information, and improve doctor–patient relationships to enhance patient satisfaction with medical services. At the macro level, government departments should work to improve the accessibility and fairness of medical services and foster harmonious doctor–patient relationships through effective policy measures. This study’s findings thus provide both theoretical support and practical directions for enhancing the quality of medical services and maximizing the social benefits of medical institutions.

## Data availability statement

The raw data supporting the conclusions of this article will be made available by the authors, without undue reservation.

## Ethics statement

This study was reviewed and approved by the Institutional Review Board of Hanghzou Normal University (approval numbers: 2019-030 and 2022-1126). All the patients provided written informed consent to participate in this study. All procedures were in accordance with the ethical standards of the responsible committee on human experimentation and with the Helsinki Declaration.

## Author contributions

YQ: Conceptualization, Methodology, Software, Writing – original draft, Validation. XW: Supervision, Validation, Writing – review & editing, Formal analysis. XH: Writing – review & editing, Software. JL: Investigation, Writing – review & editing, Data curation, Visualization. CJ: Writing – review & editing, Formal analysis. JC: Writing – original draft, Methodology. MS: Investigation, Writing – review & editing, Visualization.
